# “Figure‐of‐Eight” Suture‐Button Technique for Fixation of Displaced Anterior Cruciate Ligament Avulsion Fracture

**DOI:** 10.1111/os.12682

**Published:** 2020-04-29

**Authors:** Shi‐da Kuang, Chao Su, Xin Zhao, Yu‐sheng Li, Yi‐lin Xiong, Shu‐guang Gao

**Affiliations:** ^1^ Department of Orthopaedics, Xiangya Hospital Central South University Changsha China; ^2^ Hunan Key Laboratory of Joint Degeneration and Injury Changsha China; ^3^ Hunan Engineering Research Center of Osteoarthritis Changsha China; ^4^ National Clinical Research Center of Geriatric Disorders, Xiangya Hospital Central South University Changsha China

**Keywords:** Anterior cruciate ligament, Arthroscopy, Avulsion, Fracture, Knee

## Abstract

**Objective:**

To assess the clinical results of the “figure‐of‐eight” suture‐button technique in the arthroscopic treatment of anterior cruciate ligament (ACL) tibial avulsion fractures.

**Methods:**

This was a retrospective study reviewing data from September 2013 to June 2019. A total of 27 patients (13 males and 14 females) who underwent arthroscopic “figure‐of‐eight” suture‐button fixation for displaced ACL avulsion fractures were analyzed. The mean age of the patients in the sample was 15.8 years (10–29 years), with a mean follow‐up of 24 months (6–48 months). According to Meyers–McKeever classification, 11 patients were classified as type III and 16 as type IV. All patients were evaluated following the guidelines of the radiological union, the Lysholm knee scoring scale, and the International Knee Documentation Committee (IKDC).

**Results:**

Fractures were united within 3 months after surgery in all 27 cases. During the last follow‐up, all the anterior drawer and Lachman tests were negative, except in 1 patient whose anterior drawer test was 1° positive. The range of motion was improved from 72.22° ± 27.92° before surgery to 137.78° ± 7.38° at the last follow‐up (*P* < 0.05); the Lysholm score was improved from 45.81 ± 10.94 before surgery to 93.04 ± 5.66 at the last follow‐up (*P* < 0.05); and the IKDC score was increased from 43.89 ± 11.16 before surgery to 90.26 ± 5.86 at the last follow‐up (*P* < 0.05). In 1 patient, an inflammatory reaction was observed at the medial incision of the tibial tubercle; the symptoms disappeared with administration of antibiotics for 1 week. All patients returned to their preinjury physical activities at the last follow‐up.

**Conclusion:**

The “figure‐of‐eight” suture‐button technique achieves a satisfactory clinical outcome and provides an effective method for the treatment of displaced ACL avulsion fractures.

## Introduction

Anterior cruciate ligament (ACL) avulsion fractures or tibial eminence avulsion fractures are predominantly observed in children and adolescents because of the relative weakness of the incompletely‐ossified tibial eminence as compared with the fibers of the ACL^1^. ACL avulsion fractures of the tibial insertion are often caused by traffic accidents and sports injuries. According to Meyers and McKeever^2^, the tibial avulsion fractures can be classified into four types: minimally displaced fracture (type I), anterior elevation of the fracture fragment (type II), complete separation of the fragment from the tibia (type III), and comminution of the fragment (Type IV). Type I fractures are usually treated non‐operatively with cast immobilization, while closed reduction or arthroscopic evaluation can be attempted for the treatment of type II fractures. For displaced type III or IV fractures, open or arthroscopic reduction and fixation are generally required.

A variety of fixation methods, including Kirschner wire^3^, screw^4^, ^5^, ^6^, stainless steel wire^7^, suture^8^, ^9^, suture anchor^10^, ^11^, ^12^, and TightRope (Arthrex)‐suture button^13^ fixation have been reported, along with a series of known complications, such as non‐union after the loss of reduction, extension lag due to remaining intraarticular hardware, lesions of the physis, pain, residual laxity, and irritation and pain from retained hardware. These complications may delay the recovery process. Prior studies have compared various fixation methods for arthroscopic tibial avulsion fractures; however, there is currently no gold standard treatment^14^. The objective of the present study was to describe the arthroscopic “figure‐of‐eight” suture‐button technique for the fixation of ACL avulsion fractures and to evaluate the clinical results.

## Method

### *Inclusion and Exclusion Criteria*

The inclusion criterion was any adult patient with displaced type III or IV tibial avulsion fractures who had completed at least 6 months of clinical follow‐up. Exclusion criteria were: (i) patients with displaced type I or II tibial avulsion fractures; (ii) patients with combined ligament or meniscal injuries; (iii) patients with a history of fractures in the affected knee; and (iv) patients who suffered from moderate to severe arthritis in the affected side of the knee.

### *Patients' Information*

With approval from the institutional review board, we retrospectively reviewed the data of 27 patients who underwent arthroscopic “figure‐of‐eight” suture‐button fixation of isolated ACL tibial avulsion fractures (Meyers and McKeever's type II and III fractures) between September 2013 and June 2019. All participants had provided written informed consent. Intraoperative examination under anesthesia and diagnostic arthroscopy further confirmed eligibility for our study. The mean age of the included patients was 15.8 years, ranging from 10 to 29 years. The causes of injuries included traffic accident (19 patients), fall from height (5 patients), and slipping down (3 patients). According to Meyers–McKeever classification, 11 patients were categorized as type III and 16 patients as type IV. Plain radiographs, CT, or MRI scans were evaluated for all cases. Anterior drawer and Lachman tests were performed both preoperatively and during the last follow up. Union of fracture was defined as absence of a visible fracture line on plain radiograph. All operations in this study were performed by the same surgeon.

### *Surgical Technique*

#### *Anesthesia and Exposure*

General anesthesia was administered, with the patient being placed in the supine position. A thigh tourniquet was typically used to control bleeding and to improve visualization. The joint was fully examined through routine anterolateral and anteromedial portals. The synovial membrane, blot clots, and part of the infrapatellar fat pad were removed at 90° knee flexion. The displaced bony fragment was exposed completely. Debridement of the fibrous tissue between the fragment and the tibial bed was performed with a curette and shaver until the fragment could be reduced into the tibial bed.

#### *Reduction and Fixation*

**Figure 1:**
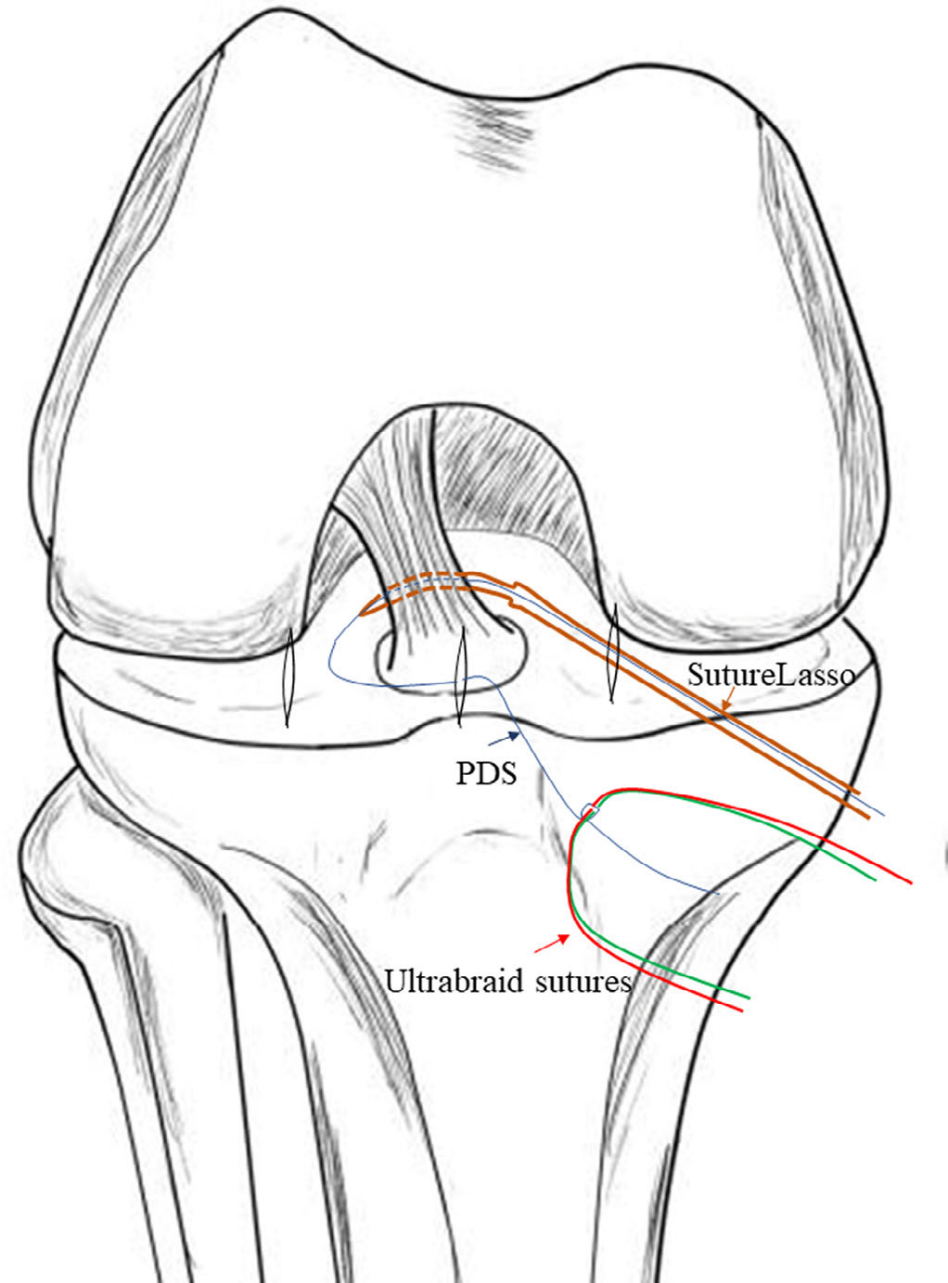
A guiding PDS (blue arrow) was passed *via* a 45° suture lasso (Arthrex) (brown arrow) from the medial side of the anterior cruciate ligament (ACL), around its back, to its posterolateral side and was used for shuttling the definitive fixation suture (UltraBraid sutures) (red arrow). Again, a second guide PDS was placed *via* a 45° SutureLasso, through the fibers of the ACL, close to its tibial bony insertion, posterior to its mid‐coronal plane, from medial to lateral.

A trans‐patellar tendon portal was established. The arthroscope was inserted through the anterolateral portal. From the anteromedial portal, a guide PDS (No. 1 polydioxanone) was placed *via* a 45° SutureLasso (Arthrex, Naples, FL), through the medial side of the ACL, around its back, and to its posterolateral side. Then the guide PDS was pulled from the medial side of the ACL out of the joint through the trans‐patellar tendon portal. Two strands of No. 2 UltraBraid sutures (Smith & Nephew, Andover, MA, USA) were tied to the lateral end of the PDS, and the medial end of the PDS was pulled out through the anteromedial portal to retrieve the UltraBraid sutures around the back of the ACL out of the anteromedial portal. Again, a second guide PDS was placed *via* a 45° SutureLasso, through the fibers of the ACL, close to its tibial bony insertion, posterior to its mid‐coronal plane, from medial to lateral. This second PDS was also pulled from the medial side of the ACL out of the joint through the trans‐patellar tendon portal. The medial ends of the UltraBraid sutures were tied to the lateral end of the second PDS, while the medial end of the second PDS was pulled out through the anteromedial portal to retrieve the UltraBraid sutures through the ACL out of the anteromedial portal (Fig. 1).

**Figure 2:**
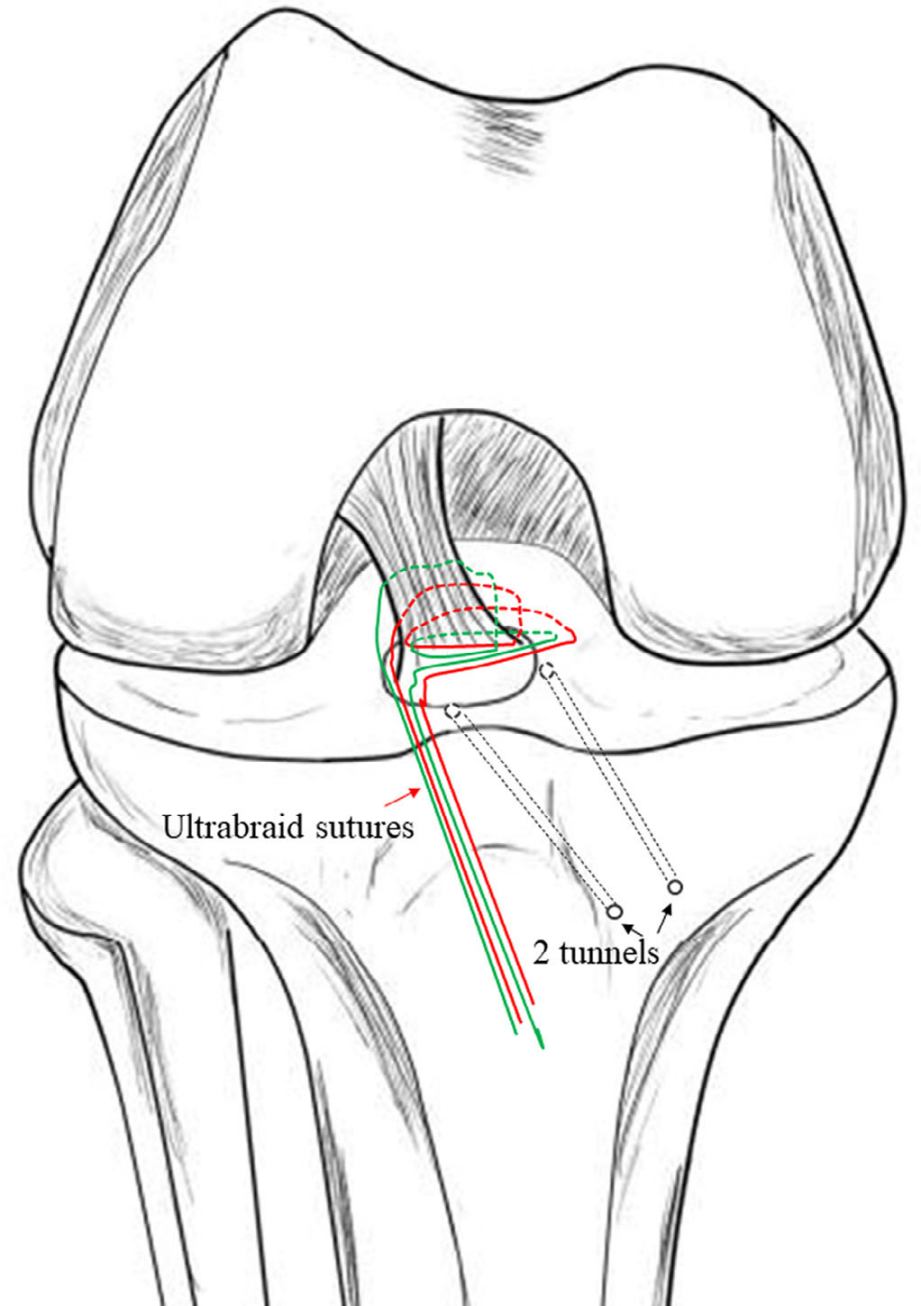
Two tunnels (black arrow) were created on the anteromedial and anterolateral sides of the tibial fracture bed with an anterior cruciate ligament (ACL) guide (Arthrex or Smith & Nephew) using a 2.4‐mm Kirschner wire.

**Figure 3:**
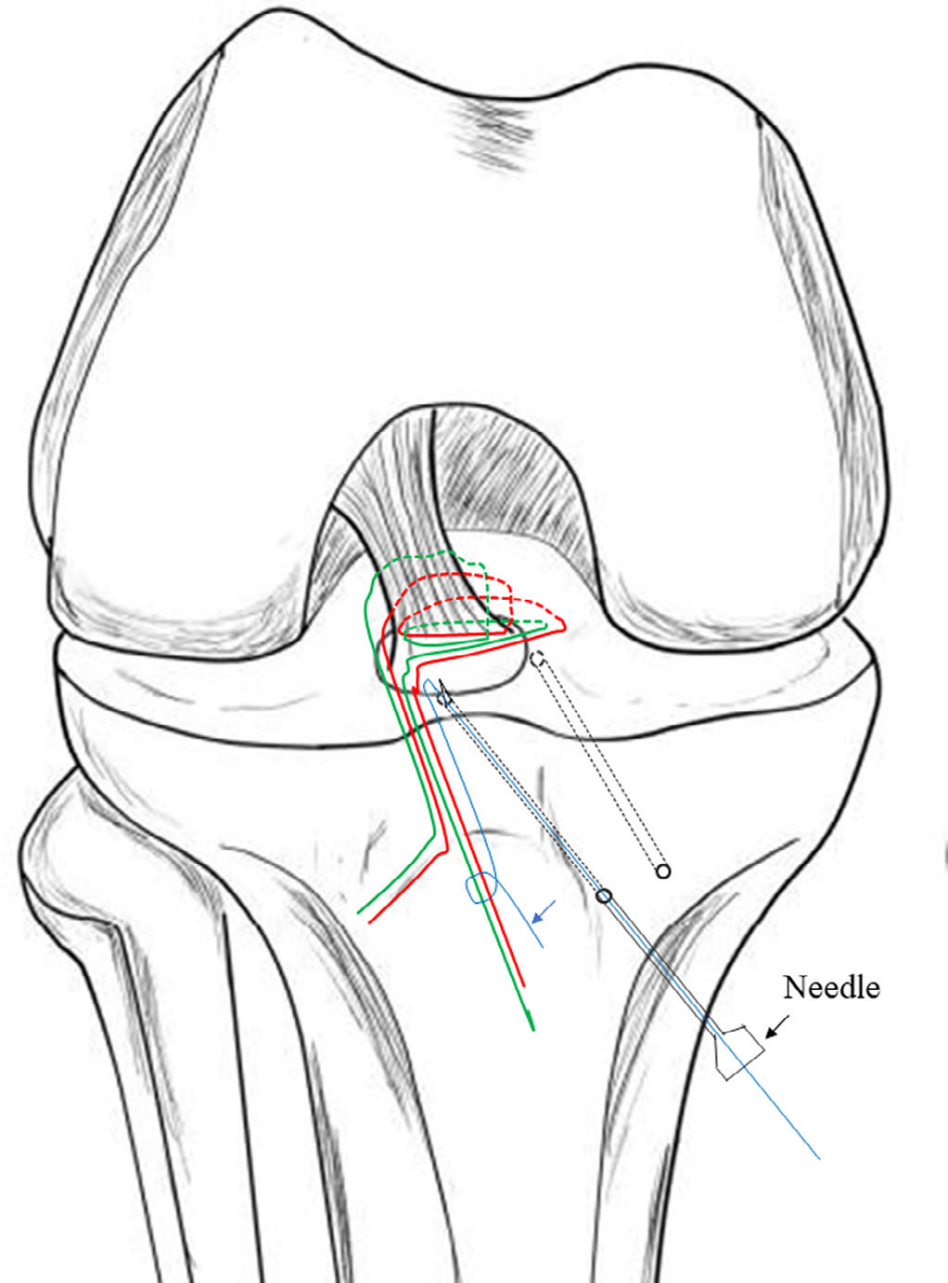
The UltraBraid sutures were pulled out through the tibial tunnel with an 18‐gauge needle (black arrow) with a loop suture made of PDS (blue arrow).

**Figure 4:**
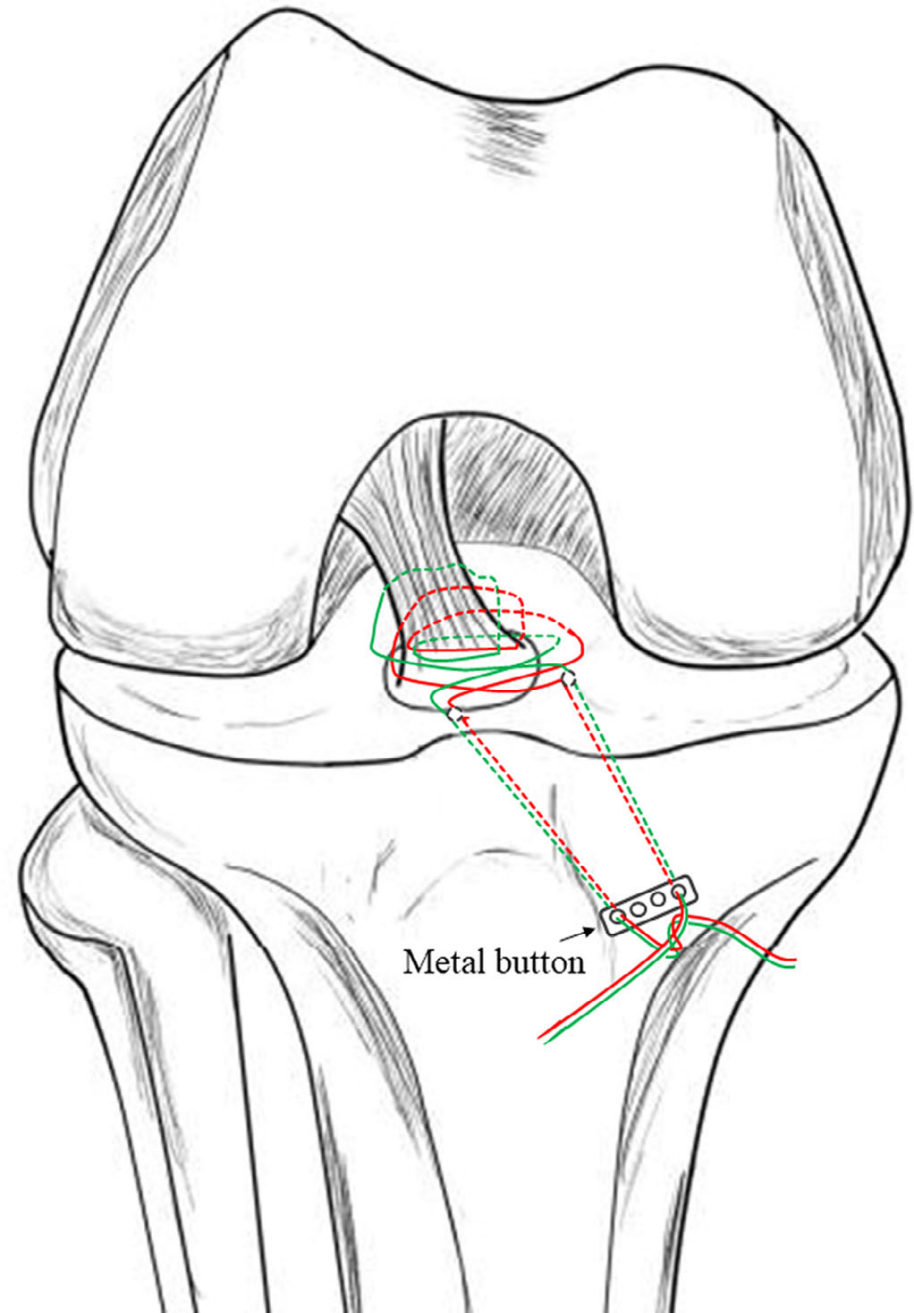
A four‐hole metal button was used for the final fixation of the UltraBraid sutures over the tibia. The lateral end of the sutures was passed through the first or second hole of the metal button. The medial end of the sutures was passed through the third or fourth hole of the button. The button was firmly secured against the anteromedial surface of the tibia and several knots were tied over it.

**Figure 5:**
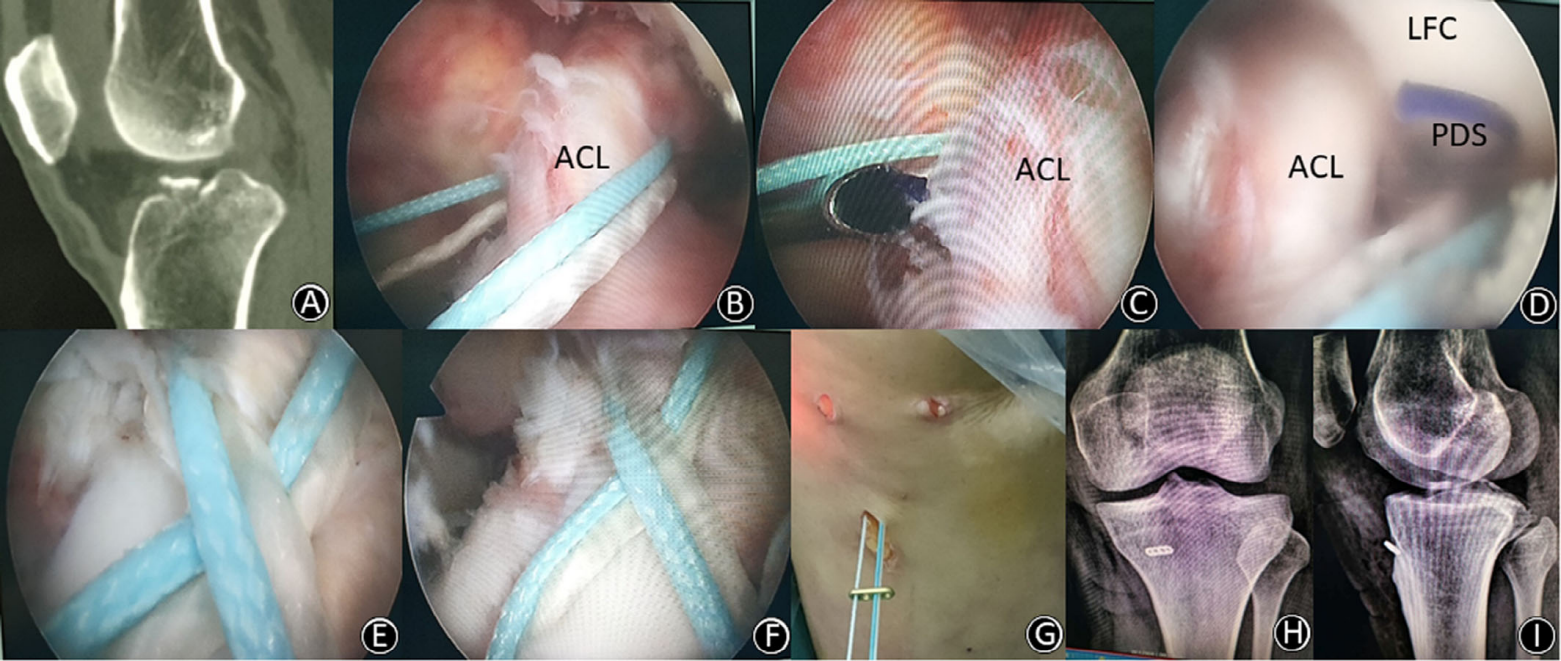
(A) A 45‐year‐old woman suffered a displaced anterior cruciate ligament (ACL) avulsion fracture (sagittal CT image) of her left knee caused by a road‐traffic accident. (B) UltraBraid sutures were passed from the medial side of the ACL, around its back, to its posterolateral side. (C, D) A guide PDS was placed *via* a 45° SutureLasso, through the fibers of the ACL, close to its tibial bony insertion, posterior to its mid‐coronal plane, from medial to lateral. (E, F, G) UltraBraid sutures were passed through two bone tunnels (being drilled in) and tied over a button on the outer cortex of the tibia; then the figure‐of‐eight fixation was completed. (H, I) Postoperative anteroposterior and lateral plain radiographs showed anatomic reduction of the fragment.

A tibial aiming device for ACL reconstruction (Arthrex or Smith & Nephew Endoscopy) was inserted through the anteromedial portal. Then a 2‐cm incision was made at the medial side of the tibial tubercle. Through this incision, two tunnels were created from the medial side of the tibial tubercle to the anteromedial and the anterolateral side of the fracture bed, respectively, with a standard ACL guide using 2.4‐mm Kirschner wire (Fig. 2). The bone bridge was approximately 6 mm in width. A puncture needle crossed the 2.4‐mm wide tunnel. A guide PDS was placed through the puncture needle into the joint and the tip was retrieved through the trans‐patellar tendon portal using a grasper (Fig. 3). The Ultrabraid sutures were crossed just at the anterior side of the ACL, above the bony fragment. Then the suture ends from the medial side of the ACL were pulled out with the guide PDS through the lateral tunnel, and those from the lateral side of the ACL were pulled out through the medial tunnel. With consistent pulling of the sutures, the fragment was adjusted into the tibial bed. Performing passive knee range of motion (ROM) exercises from full extension to 90° flexion enhanced the reduction process. While maintaining reduction under the arthroscopic view, the sutures were tightened over the metal button with the knee in 30° of flexion (Fig. 4). We refer to this suture technique as the “figure‐of‐eight” suture, as it is visually like the shape of an “8”. For more details of the procedures followed in a typical case in our study, see Fig. 5.

### *Postoperative Management*

Isometric exercises for quadriceps and straight‐leg raising exercises were performed immediately after the operation. Full weight‐bearing was permitted immediately as tolerated. The knee was immobilized in full extension using a brace for 2 weeks after the operation; then, ROM exercises were started gradually. The ROM was limited to 0° to 90° in the third and fourth week, while >120° flexion was allowed from the end of the sixth week. At 6 weeks postoperatively, the patients moved on to the standard ACL rehabilitation protocol, with the aim of returning to physical activities at 6 months. Radiographs were obtained at 1, 3, and 6 months postoperatively. At final follow‐up, the anterior drawer and Lachman tests were used to evaluate the stability of the knee.

### *Outcome Measures*

The International Knee Documentation Committee (IKDC) evaluation form was used to detect improvement or deterioration in symptoms, function, and sports activities. The response to each item was scored using an ordinal method. The most recent version had assigned scores for each possible response printed on the questionnaire. Scores for each item were summed to give a total score (excluding item 10a). The total score was calculated as (sum of items)/(maximum possible score) × 100, to give a total score of 100. An online scoring sheet was available that provides a patient's raw score and percentile score. The item for knee function prior to knee injury was not included in the total score. Possible score range 0–100, where 100 = no limitation with daily or sporting activities and the absence of symptoms.

The Lysholm knee scoring scale was also used to evaluate outcomes of knee ligament surgery, particularly symptoms of instability. The scale included eight items: limp, support, locking, instability, pain, swelling, stair climbing, and squatting. Each possible response to each of the eight items had been assigned an arbitrary score on an increasing scale. The total score was the sum of each response to the eight items, with a possible score of 100.

### *Statistical Analysis*

Statistical analyses were performed using SPSS software version 22.0 (SPSS, Chicago, IL, USA). The paired *t‐*test was conducted to test for the differences in scores between preoperative and postoperative measurements. A *P*‐value <0.05 was considered statistically significant.

## Results

### *General Results*

The 27 patients were followed up for 24 months on average (range, 6 to 48 months; mean, 24 months). The surgical duration was 30–100 min, with a mean of 68 min, and average blood loss was 30 mL (range, 10–50 mL). The total days in hospital were 2.56 days (range, 1–4 days). The average postoperative hospital stay for the arthroscopic surgery group was 1.18 days (range, 1–3 days). Analysis of the radiographs showed that the fractures healed in all cases within 3 months after surgery, with no displacement of the implants. No radiographs showed joint space narrowing or degenerative changes at the last postsurgical follow‐up.

### *Complication*

No wound infection and neurovascular injuries were observed. In 1 patient, inflammatory reaction was observed at the medial incision of the tibial tubercle. The symptoms disappeared with administration of antibiotics for 1 week.

### *Clinical Improvement*

At the last postsurgical follow‐up, the anterior drawer and Lachman tests of all patients were negative, except for 1 patient who had a 1° positive anterior drawer test result. All patients had returned to their preinjury physical activities by the last follow‐up and were satisfied with the outcome of the operation.

### *Functional Evaluation*

The ROM (Table 1) was improved from 72.22° ± 27.92° before surgery to 137.78° ± 7.38° at the last follow‐up (*P* < 0.05). The Lysholm scores (Table 1) before surgery and at the last follow‐up were 45.81 ± 10.94 and 93.04 ± 5.66, respectively (*P* < 0.05). The IKDC score (Table 1) was increased from 43.89 ± 11.16 before surgery to 90.26 ± 5.86 at the last follow‐up (*P* < 0.05).

**Table 1:** Clinical results of the suture‐button technique (mean ± SD)

Parameter	Preoperative	Last follow‐up	*P*‐value
ROM	72.22° ± 27.92°	137.78° ± 7.38°	<0.05
Lysholm score	45.81 ± 10.94	93.04 ± 5.66	<0.05
IKDC	43.89 ± 11.16	90.26 ± 5.86	<0.05

IKDC, International Knee Documentation Committee; ROM, range of motion.

## Discussion

### *General Treatment for Displaced Anterior Cruciate Ligament Avulsion Fracture*

All our patients with displaced ACL avulsion fractures achieved excellent functional and stability outcomes after avulsion fracture fixation, with a mean follow up of 24 months. To date, ACL treatments for many displaced tibial avulsion fractures have been reported to yield poor clinical results, either with limited knee extension or slight anterior knee instability^15^, ^16^, ^17^, ^18^. The main causes of these unfavorable results are improper reduction and unstable fixation in the reduction of ACL tension. Meanwhile, long‐term postoperative restrictions are also considered as a risk factor for loss of knee motion. Given that the need for postoperative restrictions depends significantly on the initial strength at the fixation site, firm fixation of the ACL avulsion fragment is crucial for mitigating motion complications.

The choice of treatment method is primarily determined by the type of avulsion fracture. The characteristics that determine management include the size, the degree of displacement, the comminution and orientation of the fracture fragment, and the integrity of the attached cruciate ligament. Avulsed fragments that are of a sufficiently big size may be treated arthroscopically, including fixation with a cannulated cancellous screw or Kirshner wire, or suture fixation. Screws may cause fracture fragment comminution during the insertion process^19^, and screws or Kirshner wires have to be removed using another procedure^19^. Screws may also cause unexpected problems if a revision surgery is required, as well as in ACL reconstruction. Bong *et al*.^20^ reported that FiberWire fixation of eminence fractures provided biomechanical advantages over screw fixation and may influence the type of treatment one chooses for patients with tibial eminence fractures. In children with small or comminuted fragments, screw fixation is usually not available. Arthroscopic pullout suture techniques should be indicated for small or comminuted fragments, although these surgical techniques are often difficult^21^. In our hospital, the arthroscopic suture‐button fixation technique is generally preferred because of the advantage of its applicability in all types of fractures.

### *Advantages of the “Figure‐of‐Eight” Suture‐Button Technique*

There are six recent reports on the use of suture fixation^1^, ^21^, ^22^, ^23^, ^24^. The discussed suture fixation methods can be fundamentally divided into two classes: one class is based on the ACL itself (ligament suture methods) and the other is based on the avulsed bone fragment (avulsed bone fragment suture methods). When the fracture of the intercondylar eminence of the tibia is comminuted or small in size, the suture methods based on the avulsed bone fragment are technically impossible, but sutures through the base of the ligament itself can provide secure fixation. Our technique, as a combination of these two methods, can be easily implemented to maintain the appropriate fixation. The figure‐of‐eight suture fixation for ACL tibial avulsion fracture is a modification of the existing traditional open surgery or arthroscopy. The small or comminuted fracture fragment can be better reduced through this novel suture fixation technique.

Another point of consideration for avulsion fracture fixation is the number of fixation points, because there are no commonly agreed criteria on this parameter. The number of fixation points ranges from 1 to 4, depending on the characteristics of the suture device and the overall technique^24^, ^25^, ^26^. The uniqueness of our technique lies in that it uses two fixation points with an excellent anatomic reduction. Further investigation into the optimal number of suture fixation points is needed.

The technique presented in this paper has not been reported previously. Its advantages (see Table 2) include anatomic reduction of the fragment, stabilization in a limited surgical time, as well as early rehabilitation with full weight‐bearing. In this study, we found that this technique is appropriate for both large and small fracture fragments, and is especially suited to comminuted fractures. In addition, it can be used in skeletally immature patients to avoid placement of hardware across the physis, which can potentially cause growth disturbance. Trans‐tibial drill tunnels cross the physis and, thus, have the potential to cause growth disturbance. However, the width of the tunnel is only 2.4 mm. The literature indicates that this carries a very small risk of causing damage to the proximal tibial physis in skeletally immature persons^27^.

**Table 2:** Advantages and limitations of the suture‐button technique

Advantages
The technique is suitable for all types of displaced ACL avulsion fracture.
No intra‐articular hardware is used.
The suture passing instrument can easily pass sutures through the ACL.
The combination of two methods (ligament suture method and avulsed bone fragment suture method) can provide proper reduction and stable fixation.
Use of a PDS loop facilitates suture retrieval through the ACL or tunnel.
A less invasive option for skeletally immature patients (only two 2.4‐mm wide tunnel).
Tunnel drilling does not need to pass through the fracture fragment.
Button reduces the cutting effect of the suture on the bone edge.
Minimal risk of wound healing complications.
The technique is effective and economical with a shorter operative time.
Limitations
The technique is technically demanding.

ACL, anterior cruciate ligament.

### *Limitations*

Several limitations of the present study deserve mentioning. First, our analysis was limited by the small number of cases and the non‐comparative single‐arm reports of surgical results. Second, the mean follow‐up term is relatively short (the maximum follow‐up period was 48 months). It is critical to include subjects with longer follow‐up periods to better evaluate growth disturbances. Third, although the button can be taken out under local anesthesia, this procedure is still associated with certain risks, such as infection.

### *Conclusion*

In conclusion, our “figure‐of‐eight” suture‐button technique using two 2.4‐mm transtibial tunnels is an easy‐to‐operate and effective method for the treatment of displaced ACL avulsion fractures, with excellent anatomic reduction and clinical results.
